# Non-Structural Proteins from Human T-cell Leukemia Virus Type 1 in Cellular Membranes—Mechanisms for Viral Survivability and Proliferation

**DOI:** 10.3390/ijms19113508

**Published:** 2018-11-08

**Authors:** Elka R. Georgieva

**Affiliations:** Department of Chemistry and Chemical Biology, Cornell University, Ithaca, NY 14853, USA; erg54@cornell.edu; Tel.: +1-607-255-4980

**Keywords:** T-cell leukemia virus type 1, p8^I^ protein, p12^I^ protein, p13^II^ protein, virus-host interactions, viral non-structural proteins, cellular membranes

## Abstract

Human T-cell leukemia virus type 1 (HTLV-1) is the causative agent of illnesses, such as adult T-cell leukemia/lymphoma, myelopathy/tropical spastic paraparesis (a neurodegenerative disorder), and other diseases. Therefore, HTLV-1 infection is a serious public health concern. Currently, diseases caused by HTLV-1 cannot be prevented or cured. Hence, there is a pressing need to comprehensively understand the mechanisms of HTLV-1 infection and intervention in host cell physiology. HTLV-1-encoded non-structural proteins that reside and function in the cellular membranes are of particular interest, because they alter cellular components, signaling pathways, and transcriptional mechanisms. Summarized herein is the current knowledge about the functions of the membrane-associated p8^I^, p12^I^, and p13^II^ regulatory non-structural proteins. p12^I^ resides in endomembranes and interacts with host proteins on the pathways of signal transduction, thus preventing immune responses to the virus. p8^I^ is a proteolytic product of p12^I^ residing in the plasma membrane, where it contributes to T-cell deactivation and participates in cellular conduits, enhancing virus transmission. p13^II^ associates with the inner mitochondrial membrane, where it is proposed to function as a potassium channel. Potassium influx through p13^II^ in the matrix causes membrane depolarization and triggers processes that lead to either T-cell activation or cell death through apoptosis.

## 1. Introduction

Human T-cell leukemia virus type 1 (HTLV-1) is the etiological agent of adult T-cell leukemia/lymphoma (ATLL) and HTLV-1-associated myelopathy/tropical spastic paraparesis (HAM/TSP) [1,2,3,4]. HTLV-1 almost exclusively infects CD4^+^ T cells, which are key in the modulation of immune responses to pathogens and tumor cells [5,6]. ATLL affects the blood, central nervous system, bone, and other visceral sites [7,8] and is an aggressive and invariably fatal disease with a survival time of less than 1 year [2,9,10]. HAM/TSP is an irreversibly progressive neurological disease characterized by demyelinating lesions in the brain and spinal cord. These lesions result in motor disorders and a low quality of life [11,12,13]. HTLV-1 is also associated with infective dermatitis and Sjögren’s syndrome (immune and endocrine-metabolic disorders), as well as thyroiditis and other diseases [10,14].

HTLV-1 was the first human retrovirus discovered as a result of extensive studies to identify the causative agent(s) of ATLL [1,15,16]. It was first isolated from a patient with cutaneous T-cell lymphoma [16]. HTLV-1 can be transmitted through biological fluids [17,18,19]. Other retroviridae family members, which are HTLV-2 [20], HTLV-3 [21], HTLV-4 [22], and HIV [23], were subsequently identified. Of these, only HTLV-2 can cause neurological disorders, some of which resemble HAM/TSP—although with a very low probability [24,25]—and immortalize normal human peripheral blood cells via co-cultivation with pre-infected donor cells in a manner similar to that of HTLV-1 [15,26,27]. Strikingly, among all HTLVs, only HTLV-1 causes malignant transformations in T cells in vivo, which is indicative of its unique mechanisms of infection leading to ATLL [28].

Worldwide, an estimated 10–20 million people are infected with HTLV-1 [29,30], although a recent study reports a smaller number of 5–10 million [31]. Among HTLV-1 carriers, 5–10% develop either ATLL or HAM/TSP [32,33,34]. Despite the devastating and often fatal impact of these viruses on human health, no means of controlling the spread of HTLV-1 or curing its associated diseases have been developed. The understanding of the mechanisms used by the virus to preclude its recognition and destruction by natural killer (NK) cells and cytotoxic T cells responsible for the control of viral infections in the body is largely insufficient [4,35,36,37]. HTLV-1 infection is usually asymptomatic, and the early assumption was that it could remain latent for decades [4,38]. However, chronically active cytotoxic T-cell responses to HTLV-1 antigens have been detected in all infected individuals, which suggests that the virus is not completely latent [34,39].

It is currently thought that in this continuing asymptomatic state, only the HTLV-1 plus-strand is latent, whereas the transcription of the minus-strand is active, resulting in the low-level expression of the HTLV-1-encoded protein basic leucine zipper factor (HBZ) [34]. Low concentrations of HBZ and its reduced affinity to the free major histocompatibility complex class I (MHC-1) preclude the immune response to the virus [40]. HTLV-1 functioning in the cell requires the expression of plus-strand-encoded proteins, which have essential roles. The factors regulating the transcription of HTLV-1 plus-strand and the interplay between the plus- and minus-strand transcriptions are not well understood. One possibility is that certain conditions, such as those in bone marrow, lymph, and lymph nodes, are optimal for virus activation and viral protein expression [34,41]. Owing to these uncertainties and deficiencies in both understanding of HTLV-1 physiology in the host and knowledge of factors triggering ATLL, HAM/TSP, and other HTLV-1-associated diseases, the cellular and molecular mechanisms of these diseases are poorly understood.

Specific interactions of the virus-encoded proteins with cellular components that modify cellular function and communication through signaling are critical for the adaptation and survival of the virus. Therefore, understanding of the functional mechanisms of proteins vital to the virus would allow for interventions affecting HTLV-1 infectivity and pathogenesis, guiding the development of viral protein inhibitors that could restrict the effects of viral proteins on cellular mechanisms and possibly restore cellular homeostasis. To this end, special attention should be paid to the HTLV-1-encoded non-structural proteins (NSPs), which are also known as “regulatory” or “accessory” proteins: p12^I^, p8^I^, p30 ^II^, p13^II^, Rex, Tax, HBZ, and HBZ-SP1(SP2) [28]. These proteins are absent in the mature virions, but are expressed in the host cell, where they act by modifying signaling pathways and the permeability of cellular membranes, redistributing ions in cellular compartments, promoting the transcription of viral proteins, inhibiting DNA repair, and ultimately altering host cell homeostasis [28,42,43,44,45,46,47]. Thus, NSPs are versatile tools that aid HTLV-1 in escaping and disabling host immune responses, optimizing viral replication and proliferation, and in some cases immortalizing infected cells. These functions make NSPs one of the primary targets for the development of therapeutics to control HTLV-1 infection and HTLV-1-linked diseases.

This review focuses on the function of HTLV-1-encoded NSPs p12^I^, p8^I^, and p13^II^ in the membranes of infected cells. The p12^I^ and p8^I^ proteins reside in the endomembranes and plasma membrane, respectively, and are critical for the adaptation, survivability, and proliferation of the virus. p13^II^ associates with and self-aggregates in the inner mitochondrial membrane (IMM) to form a potassium (K^+^) channel. Through its channel activity, p13^II^ supports HTLV-1 survivability and proliferation in the host. The current knowledge and proposed mechanisms of these proteins’ functions in the membranes of infected cells are summarized.

## 2. p12^I^ and p8^I^ Proteins and Their Roles in HTLV-1 Adaptation and Proliferation in the Host

**Figure 1:**
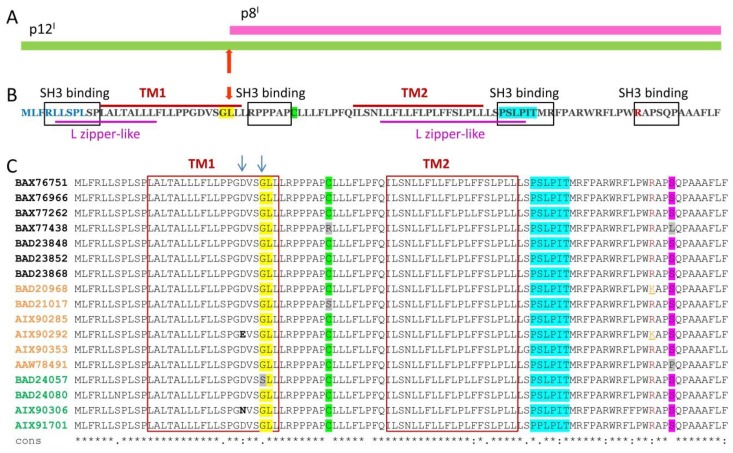
p12^I^ and p8^I^ proteins’ organization: (**A**) p8^I^ is a proteolytic product of 12I; the proteolytic cleavage site G29/L30 is indicated with an arrow; (**B**) aa sequence and putative domain architecture of full length p12^I^ are shown: The endoplasmic reticulum (ER) retention N-terminal sequence is in blue; The transmembrane helices TM1 and TM2 are designated with red bars above the sequence; SH3 binding motifs are in black rectangles; L zipper-like motifs are underlined in magenta; R88 is in red; the G29/L30 cleavage site is highlighted in yellow and indicated by a red arrow. (**C**) Alignment of multiple aa sequences of p12^I^ from randomly selected HTLV-1 strains, which were isolated from human carriers: Protein identification numbers in black, orange and green are from patients with adult T-cell leukemia/lymphoma (ATLL), patients with HTLV-1-associated myelopathy/tropical spastic paraparesis (HAM/TSP) and asymptomatic carriers, respectively. Conserved aa are indicated with asterisks under the sequences; residues D26 and G29 and their substitutions that are relevant to the level of p12^I^ and p8^I^ co-expression, are indicated with arrows; the cleavage site G29/L30, C39, the calcineurin binding motif, and residue S91, which is frequently mutated to other aa (mostly to P91), are highlighted in yellow, green blue, and magenta respectively; TM1 and TM2 are in red rectangles. The substitutions G29S, C39S, C39R, S91L, and S91P are highlighted in grey; R88 and K88 are in red and orange, respectively. T-COFFEE Multiple Sequence Alignment software was used.

Open reading frame I (*ORF-I*) of the HTLV-1 genome encodes the p12^I^ protein. p8^I^ is derived from p12^I^ after a proteolytic cleavage at position G29/L30 (Figure 1A,B). These two protein forms, p12^I^ and p8^I^, have molecular weights of ca. 12 kDa and 8 kDa and lengths of 99 amino acids (aa) and 70 aa, respectively. Both proteins are highly hydrophobic, and both reside and function in the cellular membranes although with different localization. p12^I^ resides in the membranes of the endoplasmic reticulum (ER) and cis-Golgi apparatus, whereas upon removal of the non-canonical ER retention/retrieval signal sequence in the N-terminal region of p12^I^ (Figure 1B) [48,49], p8^I^ traffics to the plasma membrane, where it is found in lipid rafts at the immunological synapse [48,50].

*ORF-I* knockout viruses are not infectious in non-human primates [51], which points to the significant roles of *ORF-I* encoded proteins. Rabbits infected with a p12^I^-deficient molecular clone of HTLV-1 showed reduced viral infectivity compared with those infected with a p12^I^-encoding clone [52]. p12^I^ is expressed early after viral entry into the host cell and is essential for maintaining infection [52,53]. Multiple critical roles of p12^I^ and p8^I^ in maintaining and spreading the virus in host organisms have been reported.

p12^I^ has two predicted transmembrane (TM) helices, TM1 and TM2 (Figure 1B,C) [49], with N-and C-termini located on the cytoplasmic side [49]; four SRC homology 3 domain (SH3) binding motifs (PXXP) [54], which are important for interactions with other proteins involved in intracellular signaling [55,56]; and leucine (L) zipper-like regions, through which the protein forms dimers in membranes [57]. Some studies have found that p12^I^ dimerization is due to the formation of a disulfide bond through the conserved cysteine residue at position 39 (C39) (Figure 1); when this residue is palmitoylated, the protein remains monomeric [57,58]. C39 palmitoylation has been suggested to be critical for ATLL transmission [58]. However, some HTLV-1 strains encode p12^I^/p8^I^ proteins that have a C39 substitution for serine (S39) or arginine (R39) (Figure 1B). Therefore, the precise role of this residue in p12^I^/p8^I^ assembly and function remains to be established. The presence of a lysine residue at position 88 (K88) decreases protein stability, as it is susceptible to ubiquitination, but an arginine at this position (R88) has a stabilizing effect [57]. R88 is present in p12^I^ isolated from HTLV-1 strains found in asymptomatic carriers and patients with ATLL, whereas K88 is found in some of the strains isolated from patients with HAM/TSP. Therefore, this residue might be relevant to the type of pathology caused by HTLV-1 [57].

p12^I^ (also p8^I^) is a highly conserved protein (Figure 1B). However, analysis of 834 patient-isolated HTLV-1 DNA sequences identified multiple aa substitutions among p12^I^/p8^I^ homologues of various HTLV-1 strains [59]. Of these, the G29S, P34L, S63P, R88K, and S91P substitutions were the most frequent mutations with possible implications for virus adaptation and proliferation in the cell. The glycine-to-serine (G29S) mutation results in the expression of non-cleavable p12^I^ [48,49,60], whereas a rare mutation of aspartic acid (D) in position 26 to either asparagine (N) or glutamic acid (E) results in the predominant expression of p8^I^ [48]. These mutations have been exploited to assess whether p8^I^ and p12^I^ expression is required for viral infectivity and persistence in macaques inoculated with B-cell lines that were transfected with HTLV-1 molecular clones carrying G29 and D26 aa’s, as well as mutants with either G29S or D26N substitutions [59]. No virus infectivity was observed when only the p12^I^ with G29S substitution was expressed. Furthermore, the abundance of p8^I^ alone (D26N mutant) limited viral persistence [59], and the absence of both p8^I^ and p12^I^ increased the susceptibility of HTLV-1-infected CD4+ T cells to T-killer cells [59]. These finding suggest that the synchronized expression of p12^I^ and p8^I^ is necessary for persistent HTLV-1 infection.

### 2.1. Roles and Functional Mechanisms of p12^I^ in the ER

**Figure 2:**
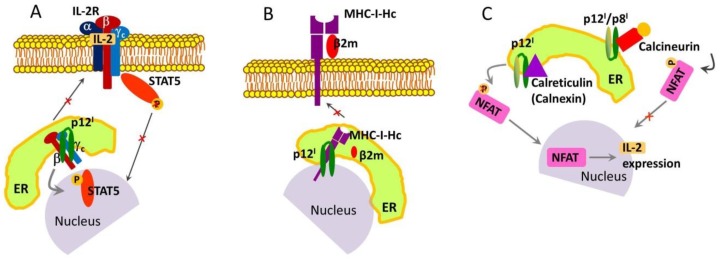
Mechanisms of p12^I^ in the endoplasmic reticulum (ER) of infected cells: (**A**) p12^I^ binds to the immature forms of interleukin-2 receptor (IL-2R) β and γc subunits preventing their traffic to the plasma membrane and the assembly of IL-2/IL-2R complex, and hence, inhibits signal transducer and activator of transcription 5 (STAT5) activation. However, upon binding to p12^I^, the β and γc subunits co-localize and provide new way for STAT5 phosphorylation and activation. Thus, p12^I^ re-routes the STAT5 signaling pathway. (**B**) Through binding to the free human MHC-I heavy chain (MHC-I-Hc), p12^I^ obliterates the assembly of functional MHC-I-Hc–β2m complex in the plasma membrane resulting in suppression of immune response. (**C**) p12^I^ has a mechanism to increase the concertation of Ca^2+^ in the cytoplasm while depleting Ca^2+^ from the ER, which affects the Ca^2+^ signaling in the cell. To do so, p12^I^ interacts with host proteins (calreticulin and calnexin), which modulate Ca^2+^ storage. As a result, dephosphorylated nuclear factor of T cells (NFAT) migrates to the nucleus and induces IL-2 expression and T-cell activation. Other possible role is that p12^I^ interacts with calcineurin, thus inhibiting NFAT.

p12^I^ enhances T-cell growth and proliferation in an interleukin-2 (IL-2)-independent manner [61,62]. IL-2 promotes T-cell proliferation and controls T-cell immune responses through the downregulation of signaling cascades [63]. These functions of IL-2 are directly dependent on its association with the IL-2 receptor (IL-2R), which is composed of three subunits: Alpha (α), beta (β), and gamma (γ_c_). In the plasma membrane, the initial binding of IL-2 to the IL-2R α-subunit further recruits the β and γ_c_ subunits to form a tertiary IL-2/IL-2R complex [64]. Co-immunoprecipitation experiments have provided evidence that p12^I^ binds specifically to the IL-2R β and γ_c_ subunits; however, the binding occurs exclusively with the immature forms of the subunits in the pre-Golgi compartments [62]. Thus, p12^I^ has an immunosuppressive role in that it prevents the maturation and trafficking of the β and γ_c_ subunits to the cell surface and thus inhibits the formation of a functional IL-2/IL-2R complex (Figure 2A). In doing so, p12^I^ also redirects the signaling pathway of T-cell activation. On the plasma membrane of uninfected T cells, the β-γ_c_ heterodimer recruits and activates the signal transducer and activator of transcription 5 (STAT5) protein, resulting in the expression of IL-2 and the activation and proliferation of T cells [65]. However, this pathway is restricted in HTLV-1-infected cells expressing p12^I^. Nonetheless, the co-localization of the β and γ_c_ subunits upon binding to p12^I^ also enhances STAT5 phosphorylation, leading to its activation and providing an efficient tool for viral control of T-cell proliferation without the need for IL-2 [61] (Figure 2A). This could be a mechanism of oncogenesis given that studies have established that STAT5 is activated in 70% of ATLL primary cells [66]. Moreover, activated STAT proteins are hallmarks of other cancers [67,68].

p12^I^ binds to the free human MHC-I heavy chain (MHC-I-Hc), as established in another co-immunoprecipitation assay [69]. The MHC-I complex plays a critical role in immunity: In infected cells, it binds peptide fragments derived from pathogens (e.g., viruses and bacteria) and displays them on the cell surface for recognition by T cells [70,71]. As a result, the T cells are activated and eradicate the infected cells. The MHC-I complex is composed of a transmembrane glycoprotein Hc, the β2-microglobulin (β2m) [71]. p12^I^ has been found to associate with MHC-I-Hc but not with the entire MHC-I-Hc–β2m complex [69] (Figure 2B). Furthermore, it has been suggested that in the ER, p12^I^ binds to a form of MHC-I-Hc that is not fully matured (most likely a less glycosylated form per the results of an electrophoresis assay) and obliterates the formation of the functional MHC-I-Hc–β2m complex [69]. Preventing the T-cell recognition of invaded somatic cells in infected individuals may be a mechanism through which HTLV-1 suppresses immune response.

Another role of p12^I^ is to downregulate intercellular adhesion molecules I and II (ICAM-1 and ICAM-2) [46], which are ligands that activate the cytotoxic response of NK cells. As a result, NK cells cannot destroy HTLV-1-infected CD4^+^ T cells even though they show reduced surface expression of the MHC-I complex and therefore constitute a target of NK cells [46]. Thus, in addition to using the mechanism of suppressing MHC-I-mediated immune response, HTLV-1 may have evolved a means of making infected cells unsusceptible to NK-cell-controlled cytolysis. Another possibility is that through ICAM-1 downregulation p12^I^ obstructs T-cell activation, since ICAM-1 is a signaling molecule in this process [72]. However, the exact physiological effect of ICAM-1 downregulation by p12^I^ is unknown, because ICAM-1 expression is enhanced in Tax-producing cells [73], as well as in HTLV-1-positive cell lines and ATLL cells from patients [74] that might minimize the effect of p12^I^.

Other studies have suggested that the p12^I^ protein increases the concentration of calcium ions (Ca^2+^) in the cytoplasm and concurrently reduces the amount of Ca^2+^ available for release from the ER, thereby enhancing T-cell activation by modulating Ca^2+^ signaling [75]. Increased cytosolic Ca^2+^ activates the phosphatase calcineurin, which dephosphorylates the nuclear factor of T cells (NFAT) [76]. Dephosphorylated NFAT migrates to the nucleus and induces the expression of IL-2 [77], which leads to T-cell activation. A possible mechanism of p12^I^-induced cytosolic Ca^2+^ increase is through binding to the host proteins [78], which modulate Ca^2+^ storage, such as calreticulin and calnexin [79] (Figure 2C). This hypothesis is supported by the observation that p12^I^-induced NFAT activation is decreased in cells co-transfected with p12^I^ and increased doses of calreticulin [75]. Furthermore, the activation of Ca^2+^ influx channels in the plasma membrane contributes to the increase in cytosolic Ca^2+^ [75]. On the contrary, another possible role of p12^I^ may be to inhibit the function of NFAT through tight binding to calcineurin (Figure 2C), thereby leading to T-cell inactivation—p12^I^ has a calcineurin-binding motif in its C-terminal soluble domain (Figure 1B,C), which is homologous to the calcineurin-binding motif in NFAT [80]. Thus, similar to other proteins, such as the myocyte-enriched calcineurin-interacting proteins and A238L from African swine fever virus [81,82], p12^I^ might act as a negative regulator of NFAT and possibly other calcineurin-mediated physiological processes by binding to calcineurin. However, because p12^I^ and p8^I^ share the same C-terminal sequence located in the cytosol (Figure 1) [49], it is currently unclear whether only one of them or both interact with calcineurin. Furthermore, because calcineurin regulates protein activity through protein–protein interactions and phosphatase activity (62, 63), p12^I^ (and p8^I^) may be a substrate of calcineurin if it undergoes phosphorylation/dephosphorylation in vivo, which has yet to be determined (64). It should also be mentioned that at present, there is no clear understanding of how and under what conditions p12^I^ acts as a T-cell activator or inactivator by modulating Ca^2+^ levels and calcineurin activity, thus regulating the NFAT phosphorylation state. The dual role of p12^I^ in T-cell activation/inactivation is similar to that of Bcl-2 protein when it resides in the ER membrane, the mechanism of which is also unresolved [83,84].

### 2.2. Roles and Functional Mechanisms of p8^I^ in the Plasma Membrane

**Figure 3:**
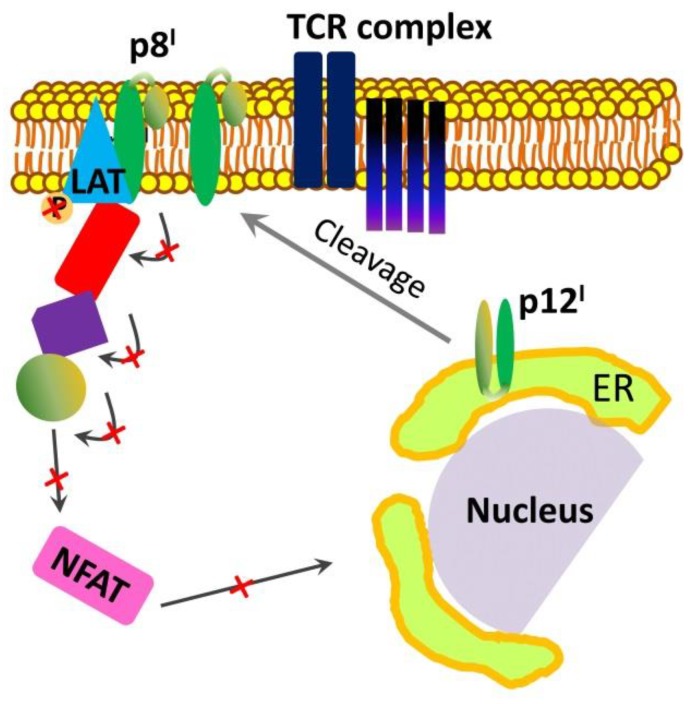
p8^I^ inhibits T cell receptor (TCR) signal transduction and NFAT activation: p8^I^ is a proteolytic product of p12^I^ in the ER membranes, the protein traffics to plasma membrane and localizes in the immunological synapse; p8^I^ interacts with and inhibits the phosphorylation of the transmembrane protein called linker for activation of T cells (LAT) and by doing so, p8^I^ disables LAT to transmit signals from TCR and to interact with other proteins, such as phospholipase C gamma 1, Vav proteins, and lymphocyte-specific protein tyrosine kinase (shown in red, cyan and green) on the TCR signaling pathway.

p8^I^ may provide another avenue to T-cell inactivation. It has been found that p8^I^ downregulates the transduction of proximal T-cell receptor signaling [48,50]. When localized at the immunological synapse in the plasma membrane, p8^I^ inhibits the signaling cascade leading to T-cell activation when in contact with antigen-presenting cells [85,86]. To do so, p8^I^ interacts with the protein called linker for activation of T cells (LAT) and decreases LAT phosphorylation, which results in decreased phosphorylation of the downstream T-cell signaling proteins phospholipase C and Vav and downregulation of NFAT activity (Figure 3) [60]. In this way, p8^I^ favors viral persistence by inhibiting the immune responsiveness of T cells. Conclusions about these p8^I^ activities were made based on a study in Jurkat T cells expressing p8^I^ and an analysis of proteins and their post-translational modifications in cell lysates [60]. However, at the time of the study, nothing was known about p8^I^ as a cleavage product of p12^I^, which migrates to the cell membrane. Also, the study was conducted using cells transfected with the precursor p12^I^ protein. Therefore, the authors believed that p12^I^ was responsible for the observed effects.

**Figure 4:**
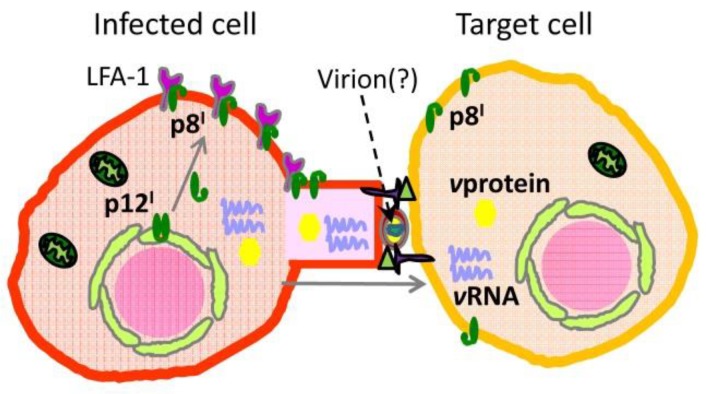
p8^I^ provides a mechanism for virus transmission through increased conduit formation: p8^I^ interacts with and enhances the clustering of lymphocyte function–associated antigen-1 (LFA-1) in the plasma membrane, and by doing so facilitates the contact between T cells. This results in the formation of conduits between infected and healthy cells, resulting in virus transmission. It is currently unknown whether this transmission occurs through a virion assembly.

Further research found that in the plasma membrane, the activities of p8^I^ protein increase the number and length of cellular conduits, as well as the likelihood of virus transmission [43]. These findings were established in studies of p8^I^ expressed in Jurkat T cells: p8^I^ protein co-localizes with and increases the clustering of lymphocyte function–associated antigen-1 (LFA-1) and also enhances the contact between T cells through adhesion to clustered LFA-1. Furthermore, these studies demonstrated that p8^I^ increases T-cell contact among primary lymphocytes, as observed in peripheral blood mononuclear cells (PBMCs) and MT-2 cells overexpressing p8^I^, in which conduits formed preferentially between resting PBMCs and MT-2 cells [43]. Notably, p8^I^ protein was visualized in these conduits. The increased conduit formation by p8^I^ was also confirmed in co-culturing experiments with non-transfected and p8^I^-transfected Jurkat T cells, as well as non-transfected Jurkat T cells and p8^I^-transfected MT-2 cells. A significant amount of p8^I^ was transferred to the non-transfected cells [43]. Thus, p8^I^ provides an additional mechanism for virus transmission through increased conduit formation (Figure 4) along with transfer through virological synapses [87] and “viral biofilm” formation [88]. The capability of p8^I^ to inactivate and segregate T cells, as well as to enhance the formation of cellular conduits, protects the virus from immune recognition, thereby ensuring efficient virus propagation.

The large body of predominantly in vivo studies of p12^I^ and p8^I^ proteins points to the indispensable roles of these proteins in modulating the signaling pathways of host T cells. This modulation suppresses immune responses and compromises the capability of the infected organism to identify and eliminate HTLV-1. Therefore, these proteins help the virus to establish long persistence and, in some cases, cause malignant cell transformations.

## 3. p13^II^ Protein and Its Role in the Control of Mitochondrial Apoptosis

**Figure 5:**
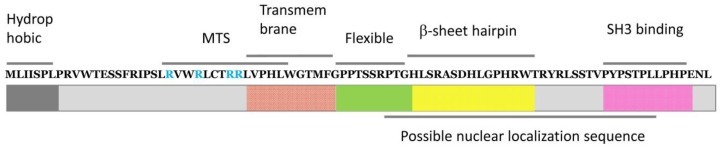
Amino acid sequence and domain architecture of p13^II^ protein: p13^II^ has hydrophobic N-terminal followed by an amphipathic helix, which contains the mitochondrial targeting sequence (MTS); transmembrane helix; highly flexible region; predicted β-sheet region; SH3-binding sequence with PXXP motif; and possibly nuclear localization sequence. The arginines (R) in MTS are colored in blue.

p13^II^ is encoded by the *ORF-II* of HTLV-1, and it corresponds to the C-terminal region of another NSP, p30^II^, which has two nucleus localization/retention sequences (NLS’s) in its N- and C-terminal regions and resides and functions in the nucleus [89,90,91]. p13^II^ might have only the C-terminal NLS (Figure 5). Initially, p13^II^ was thought to be a predominantly nuclear protein, as it was found in the nucleus of p13^II^-transfected HeLa cells [49]. Further studies demonstrated partial nuclear localization upon p13^II^ co-expression with the HTLV-1-encoded transcriptional regulator Tax protein [89,92]. In nuclear speckles, p13^II^ directly binds Tax [93], thus reducing Tax transcriptional activity and viral expression [92]. However, the role of p13^II^ as a nuclear protein is not discussed in detail herein, as it is beyond the scope of this review, which focuses on p13^II^ function in the IMM. It is currently known that p13^II^ has a mitochondrial targeting sequence (MTS), localizes in the IMM, and affects mitochondrial morphology and function [89,94,95,96,97]. A critical role for p13^II^ has been suggested by the finding that persistent viral infection failed to establish in rabbits inoculated with cells expressing p13^II^-deficient HTLV-1, but not in rabbits inoculated with cells expressing wild-type HTLV-1 [98].

p13^II^ is a positively charged 87-aa protein with multi-domain organization (Figure 5) [94,95] composed of (i) a hydrophobic N-terminus; (ii) an arginine-rich amphipathic α helix comprising residues 20–30 and including the MTS (LRVWRLCTRRLVPHL). (Note, however, that this MTS differs from the canonical MTS’s, which are located at the N-terminal and cleaved after protein insertion in the IMM.) [99]; (iii) a transmembrane region composed of residues 31–41; (iv) a highly flexible region comprising residues 42–49, which might serve as a hinge in the structure of folded p13^II^; (v) a predicted β-sheet hairpin region encompassing residues 60–75; and (vi) a C-terminal PXXP motif, which likely interacts with SH3 domains in signaling proteins, although such activities of p13^II^ have not been reported.

p13^II^ may have an NLS as well, which might adopt a conformation favorable for trafficking the protein to the nucleus under conditions that mask the MTS. Thus, p13^II^ could fulfill several cell-compartment-dependent functions; however, currently, this multi-functional role is only hypothetical [89,100]. The protein is highly conserved among viruses from distant geographical regions [94]. After its expression in the host cell, p13^II^ is trafficked to and inserts into the IMM, forming predominantly helical self-assemblies with molecular weights higher than that of a monomer with cation channel activity [89,94,95,97,101,102]. The current view is that p13^II^ is predominantly a potassium (K^+^) channel [96,102].

It has been found that a synthetic construct of full-length p13^II^ localizes in the IMM of isolated energized rat liver mitochondria and triggers K^+^ influx that results in modified mitochondrial morphology and swelling [96]. This effect was observed at p13^II^ concentrations between 5 nM and 400 nM and could be reversed by adding a protonophore that collapses IMM potential (ΔΨ_m_). Furthermore, this study showed that p13^II^ causes IMM depolarization, which increases proton transport through the pumps of the electron transport chain (ETC) and thus restores membrane potential while increasing oxygen consumption. As a result of p13^II^-enhanced ETC activity, the level of reactive oxygen species (ROS)—which are produced in part in the mitochondria [103] and control cellular processes [104]—increased as well. Both IMM depolarization and elevated ROS concentration lead to a lowering of the threshold for the opening of the permeability transition pore [104,105,106] and ultimately triggering apoptosis. No such effects were observed for p13^II^ mutants with alanines instead of arginines in the MTS (Figure 5), which points to a key role for these positively charged residues. The importance of arginines in the MTS for p13^II^ function in the IMM has also been demonstrated in other studies in which these residues were substituted for glutamines, prolines, and leucines; by contrast, no effect on p13^II^ association with the IMM was observed [94,95].

An analogous effect of mitochondrial membrane depolarization, which is dependent on p13^II^ expression level and the presence of native arginines in the MTS, has been observed in HeLa cells [94]. Studies using p13^II^-transfected HeLa cells and Jurkat T cells transduced with a lentiviral vector expressing p13^II^ also showed enhanced ROS production [107]. However, unlike in isolated mitochondria [96], an increased ROS level was not recorded unless p13^II^ was expressed under conditions of glucose starvation [107]. The authors suggested that p13^II^ is sufficient to increase ROS levels, but at physiological glucose levels, the effect is counteracted by ROS scavengers. Strikingly, in both of the tumor cell lines, the expression of p13^II^ under glucose-deprived conditions significantly enhanced the probability of cell death (3- to 5-fold) compared with that associated with expression under conditions with normal glucose concentrations [107]. This outcome can be directly correlated to the increased level of ROS, as these species play critical roles in cell turnover [108].

**Figure 6:**
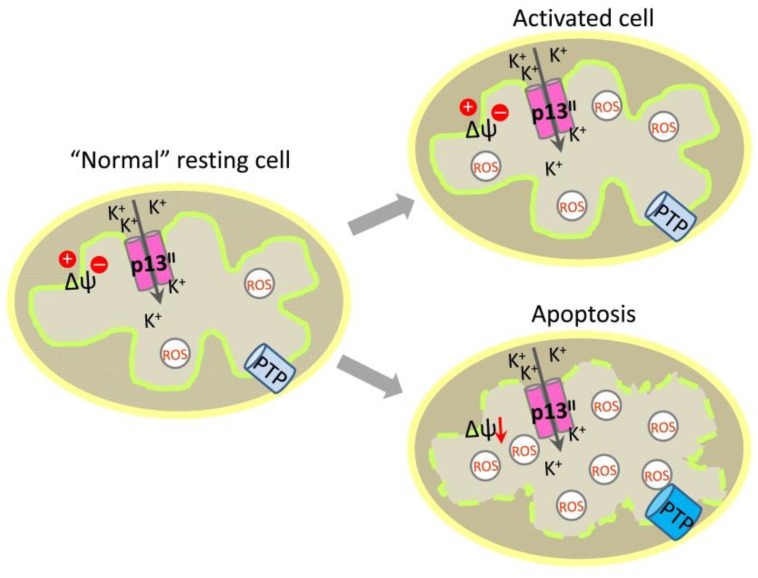
Model of p13^II^ function in T cells: p13^II^ forms oligomeric K^+^ channel in inner mitochondrial membrane (IMM); K^+^ influx affects ΔΨ_m_ and triggers processes that lead to ROS formation; at physiological ROS concertation, this leads to activation of the “normal” T cells; at very high toxic levels of ROS in transformed cells, this leads to apoptosis and cell death.

At physiological levels, ROS are signaling molecules, whereas high ROS levels trigger cell death through apoptosis. More recent studies have found that the expression of wild-type p13^II^ activates primary T cells from their resting state, a change that depends on ROS concentration and K^+^ flux [107]. In general, ROS at upper physiological limits trigger the activation of T cells, leading to their division [108]. These findings might suggest that p13^II^ protein controls the turnover of infected T cells by eliminating those that undergo cancerous transformations while activating and promoting the division of those with regular morphology. Thus, the protein might increase the number of “normal” infected cells supporting virus proliferation and long-term persistence in HTLV-1 carriers. However, at present, the exact mechanisms through which p13^II^ affects cell physiology and pathology are poorly understood. The existing view is that the protein acts by modulating levels of mitochondrial ROS [89,107]. Lacking molecular details, the current functional model of IMM-associated p13^II^ in HTLV-1-infected cells assumes that the protein inserts into the IMM to form oligomeric channels. The channel activity of p13^II^ induces a ΔΨ_m_-dependent K^+^ current and membrane depolarization. This effect is probably compensated by the enhanced activity of the ETC, leading to ROS formation. As a result, T cells undergo either activation at physiological ROS concentrations or apoptosis at toxic ROS levels (Figure 6) [96,102,107].

Alternative mechanisms for p13^II^-controlled T-lymphocyte death and survival also have been proposed. It has been suggested that p13^II^ is not an apoptotic factor per se but instead modulates other proteins on the pathway of apoptosis signaling [89,109]. One study showed that p13^II^-expressing Jurkat T cells are sensitive to caspase-dependent ceramide- and FasL-induced apoptosis [109]. Ceramide is one of the key signaling molecules in apoptosis, and its formation can be induced by several stress factors, including oxidative stress and ROS [110]. FasL, a cytokine found predominantly in the cells of the immune system, is the ligand for the Fas receptor. FasL–Fas binding triggers apoptosis; FasL expression is also induced under conditions of cell stress [111]. Moreover, a reduction in FasL-induced apoptosis has been observed in p13^II^-expressing cells after treatment with a chemical inhibitor of Ras protein, which suggests that p13^II^ might specifically alter Ras-mediated apoptosis [109]. These findings could reveal a possible apoptotic mechanism of p13^II^ that aligns well with p13^II^ channel activity in the IMM leading to ROS formation.

## 4. Roles of Tax, HBZ, and p30^II^ NSPs in HTLV-1 Life Cycle

Because this review focuses on the function of HTLV-1-encoded NSPs p8^I^, p12^I^, and p13^II^ in the cellular membranes, only a brief outline of the roles of Tax, HBZ, Rex, and p30^II^, which are not membrane proteins, in HTLV-1 functioning in the infected cells is provided. Their properties and roles in the infected cells are described in excellent reviews published elsewhere [28,34,42,47,112,113,114,115,116].

Tax is a ca. 40 kDa protein with a multi-domain organization that facilitates interactions with a range of cellular components in the nucleus and cytoplasm that result in modified cellular functions [93,112]. It regulates the expression of viral and cellular proteins; supports the proliferation of infected cells and accumulation of genetic modifications by precluding cell cycle arrest and inhibition of DNA damage repair; and induces cellular transformations. Tax is currently considered the most important protein for HTLV-1 oncogenesis [93,112,117,118].

HBZ is the only HTLV-1-encoded protein, which is expressed through the minus-strand transcription; is composed of an N-terminal activation domain, a central domain, and a basic leucine-zipper domain; and localizes in the nucleus [34,112,113]. HBZ antagonizes many of the activities of Tax, and its major functions include maintaining long-lasting asymptomatic infection, promotion of T-cell proliferation, inhibition of apoptosis and autophagy, and disrupting genomic integrity [112,113]. The protein is also implicated in all stages of ATLL progression.

Rex is a ca. 27 kDa protein with several functional domains through which it binds to messenger RNA (mRNA) and interacts with both viral and host proteins [114,115]. As an mRNA binding protein, it plays the role of a post-translational regulator of HTLV-1 mRNA: Its most important functions are to transport mRNA from the nucleus to the cytoplasm and enhance the translation of viral structural proteins [114].

p30^II^ protein is the precursor of p13^II^ but has entirely nuclear localization. It prevents the export of Tax/Rex mRNA from the nucleus to the cytoplasm, thus acting as a downregulator of Tax and Rex and suppressor of viral replication [49,116]. p30^II^ binds specifically to and forms complexes with both Tax and Rex, and these interactions are stabilized by the presence of viral mRNAs.

## 5. Conclusions

This review focuses on the roles of the HTLV-1-encoded regulatory NSPs p8^I^, p12^II^, and p13^II^, which function in the organelles and plasma membranes of infected cells. Current knowledge suggests that these proteins aid the virus in escaping immune surveillance through mechanisms that modify multiple signaling cascades and regulate the cell cycle by inducing cell proliferation or apoptosis. Thus, these proteins are critical to viral adaptation, long survival, and proliferation. In-depth understanding of their mechanisms remains to be accomplished, however. It is currently unknown how these proteins interact with cellular components. Furthermore, multiple functions have been proposed for each of the three proteins, but the factors determining which function is initiated at which viral stage, or whether multiple functions are fulfilled in parallel, are poorly understood. Therefore, along with in-cell studies, a detailed explanation of protein structure and mechanism at the molecular level would be particularly helpful. Specifically, in vitro studies of recombinantly produced purified p8^I^, p12^II^, and p13^II^, which currently are extremely limited or even nonexistent, would both provide information about the structural bases of the interactions of these proteins with host membranes and proteins and clarify how the structures and membrane environment facilitate protein functions. This knowledge would greatly expand the general understanding of HTLV-1 mechanisms and inform the development of approaches to gain control of cellular dynamics and immune responses.
